# CD40L mediated alternative NFκB-signaling induces resistance to BCR-inhibitors in patients with mantle cell lymphoma

**DOI:** 10.1038/s41419-017-0157-6

**Published:** 2018-01-24

**Authors:** Hilka Rauert-Wunderlich, Martina Rudelius, Ingolf Berberich, Andreas Rosenwald

**Affiliations:** 1 Institute of Pathology, University of Würzburg and Comprehensive Cancer Center (CCC) Mainfranken, Würzburg, Germany; 2 Institute of Pathology, University of Düsseldorf, Düsseldorf, Germany; 3 Institute of Virology and Immunobiology, University of Würzburg, Würzburg, Germany

## Abstract

Drug resistance is a significant obstacle in cancer treatment and therefore a frequent subject of research. Developed or primary resistance limits the treatment success of inhibitors of the B cell receptor (BCR) pathway in mantle cell lymphoma (MCL) patients. Recent research has highlighted the role of the nuclear factor-kappa B (NFκB) pathway in the context of resistance to BCR inhibitors in MCL. In this study, we analyzed the dependency of MCL cell lines on NFκB signaling and illustrated the ability of CD40L to activate the alternative NFκB pathway in MCL. This activation leads to independency of classical NFκB signaling and results in resistance to BCR inhibitors. Therefore, ligands (such as CD40L) and their activation of the alternative NFκB pathway have a major impact on the drug response in MCL. Furthermore, this study indicates a protective role for cells expressing specific ligands as microenvironmental niches for MCL cells and underlines the significance of therapeutically targeting alternative NFκB signaling in MCL.

## Introduction

Mantle cell lymphoma (MCL) is a rare B cell non-Hodgkin lymphoma characterized by a t(11;14)(q13;q32) translocation, which leads to *cyclin D1* overexpression^1,2^ and cell cycle deregulation^3^. In the past few years, advances have been made in treating MCL patients by targeting the B cell receptor (BCR) pathway with ibrutinib^4^. Bruton’s tyrosine kinase (BTK) inhibitor occupies the active site of BTK and therefore blocks BCR signaling^5^, which is essential to malignant B cells^6^. Unfortunately, some MCL patients show primary resistance to ibrutinib or develop secondary resistance after treatment. The reasons for primary resistance in patients are widely unknown, whereas for secondary resistance, Chiron et al. identified a C481S mutation at the ibrutinib binding site of BTK^7^. Although novel second-generation BTK inhibitors are being evaluated^8^, understanding the reasons for primary resistance and further deciphering the molecular pathology of MCL is an important topic in research.

Rahal et al. showed that some MCL cell lines resistant to the BCR inhibitors ibrutinib and sotrastaurin have mutations in players of the alternative nuclear factor-kappa B (NFκB) pathway. These mutations lead to activation of alternative NFκB signaling and identify an MCL subgroup that is independent of BCR signaling^9^. This mechanism of resistance highlights the importance of BCR and NFκB signaling in the pathogenesis of MCL^10^.

Drug resistance is a significant obstacle in the treatment of cancer patients, and microenvironmental signaling often plays a crucial role by providing individual niches for cancer cells^11^. Recently, this role of microenvironmental effects was also described in MCL^12–14^. Apart from the mentioned mutations, microenvironmental signaling can also cause activation of the alternative NFκB pathway. Therefore, we questioned whether microenvironmental activation of the alternative NFκB pathway can lead to BCR inhibitor resistance in MCL. An important ligand in microenvironmental signaling in lymphomas is tumor necrosis factor (TNF) ligand superfamily member 5 (CD40L)^15,16^. CD40L belongs to the TNF ligand superfamily, binds to TNF receptor superfamily member 5 (CD40), and has a major role in B cell proliferation and differentiation^17^ as well as an effect on lymphomagenesis^18^. CD40L can activate both the classical and the alternative NFκB pathways^19,20^. Activation of the classical NFκB pathway, induced by the binding of a ligand to its receptor, leads to activation of the IκBα-kinase (IKK) complex, which is composed of NFκB essential modifier (NEMO), IKK-α (IKK1), and IKK-β (IKK2). This active complex then phosphorylates inhibitory IκB proteins or the IκB domain (functioning as IκB proteins) containing precursors, leading to their proteasomal degradation. IκB proteins restrain NFκB transcription factor dimers in the cytoplasm, and their degradation leads to the translocation of the transcription factor to the nucleus^21–23^.

Activation of the alternative NFκB pathway by a ligand results in the accumulation of mitogen-activated protein kinase kinase kinase 14 (NIK) and the subsequent phosphorylation of NFκB subunit 2 (p100) by IKK1. This phosphorylation activates NFκB subunit 2 (p52) and V-Rel avian reticuloendotheliosis viral oncogene homolog B (RelB)-containing NFκB dimers and allows their translocation to the nucleus^21–23^. TNF receptor-associated factor (TRAF) proteins also play a major role in NFκB signaling, and TRAF2 is necessary for classical NFκB pathway activation. TRAF2, together with TRAF3, shows inhibitory functions on alternative NFκB pathway activation by forming a complex with cellular inhibitors of apoptosis, leading to the ubiquitination and proteasomal degradation of NIK^23^. Interestingly, aberrant alternative NFκB signaling reportedly contributes to the development of lymphoid malignancies^24^.

The MCL cell line MAVER-1 harbors a biallelic *TRAF3* deletion, leading to accelerated activation of the alternative NFκB pathway^9^. We and others have previously shown the sensitivity of REC-1 cells to BCR inhibitors^9,25^. In this study, we therefore compared the effects of CD40L-mediated signaling in REC-1 and MAVER-1 cells.

## Results

### MCL cell lines with genetic lesions causing elevated alternative NFκB pathway activity are less dependent on IKK2-mediated signaling

**Fig. 1:**
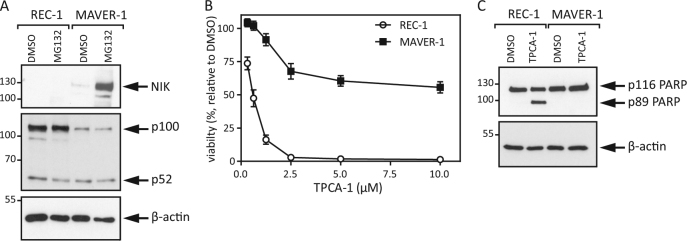
REC-1 and MAVER-1 cells show distinct dependencies on classical NFκB signaling **a** MCL cell lines were treated with MG132 (20 μM) for 8 h, and whole cell lysates were analyzed by Western blot. **b** REC-1 and MAVER-1 cells were incubated with increasing concentrations of TPCA-1 for 48 h, and viability was determined via MTT assay. **c** Whole cell lysates of the MCL cell lines were prepared after treatment with TPCA-1 (5 μM) for 24 h and were subsequently analyzed by Western blot

To analyze the effect of the TRAF3 mutation in MAVER-1 cells on the activity of the alternative NFκB pathway, we treated MCL cells with the proteasome inhibitor MG132 and detected higher levels of NIK in comparison to REC-1 cells (Fig. 1a). In addition, we detected an already higher basal level of processed p52 in relation to p100 levels in MAVER-1 cells than in REC-1 cells. This result reveals higher basal activity of the alternative NFκB pathway in MAVER-1 than in REC-1 cells. To further decipher the dependency of MCL cells on NFκB signaling, we challenged the cells with the IKK2-specific inhibitor TPCA-1, which inhibits classical NFκB signaling^26,27^. MAVER-1 cells showed only minor viability effects of TPCA-1 treatment compared to REC-1 cells, and Western blot analysis of whole cell lysates confirmed the resistance of MAVER-1 cells to TPCA-1 treatment (Fig. 1b, c). The precise inactivation of the classical NFκB pathway by TPCA-1 and the resistance of MAVER-1 cells show that these cells have, to some extent, overcome the described dependency of MCL on classical NFκB signaling^9^.

### Ligand stimulation activates the alternative NFκB pathway and rescues REC-1 cells from IKK2 inhibition-mediated toxicity

**Fig. 2:**
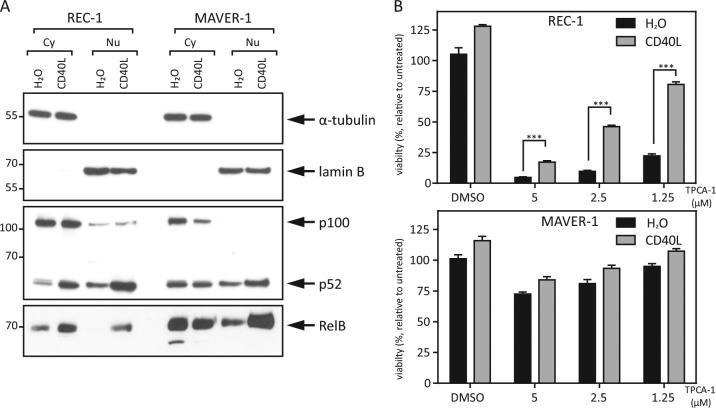
CD40L rescues IKK2 inhibition-mediated toxicity by activating the alternative NFκB pathway **a** REC-1 and MAVER-1 cells were stimulated with CD40L (100 ng/ml) or H_2_O as a control for 18 h, and cytoplasmic (Cy) and nuclear (Nu) protein fractions were analyzed by Western blot. α-Tubulin and lamin B served as controls for the purity of the individual cell compartment fractions. **b** MCL cell lines were stimulated with CD40L (100 ng/ml) or H_2_O as a control overnight followed by TPCA-1 treatment at the indicated concentration. After an additional 24 h, viability was determined by MTT assay (*p < 0.001; **p < 0.0001; ***p < 0.00001)

To show the effect of CD40L stimulation in MCL cell lines on the activity of the alternative NFκB pathway, we treated REC-1 and MAVER-1 cells with CD40L overnight and analyzed the cellular localization of NFκB transcription factors. CD40L treatment caused accelerated p100 processing to p52 and a higher expression of the active transcription factors p52 and RelB in the nucleus in REC-1 cells. This effect was also detectable in MAVER-1 cells, but to a lesser extent (Fig. 2a). These data reveal and underline the described ability of CD40L to activate the alternative NFκB pathway in MCL cells^20^.

The cell line REC-1 showed a high sensitivity towards treatment with the IKK2-specific inhibitor TPCA-1 (Fig. 1b, c and  2b), yet prestimulation with CD40L significantly rescued REC-1 cells from TPCA-1-induced cell death (Fig. 2b). This effect was considerable compared to the basal enhanced proliferation after CD40L prestimulation in the control group. In the TPCA-1-resistant MAVER-1 cell line, CD40L also slightly enhanced proliferation but with no significant effect. Taken together, these results illustrate the ability of CD40L to activate the alternative NFκB pathway in MCL cell lines and to substitute the dependency on classical NFκB signaling in REC-1 cells through the activity of alternative NFκB signaling. Chiron et al. argued for a functional role of the anti-apoptotic protein Bcl-xL in the context of CD40 stimulation in some MCL cell lines^20,28^. We measured the Bcl-xL expression level and show in Supplementary Figure 1 that there was no major difference in Bcl-xL expression in REC-1 cells. However, compared to MAVER-1 cells, REC-1 cells showed a very high basal Bcl-xL expression level. In MAVER-1 cells, we observed faint induction of Bcl-xL, as described by Chiron et al. which was inhibited by TPCA-1. Therefore, in this cell line, the classical NFκB pathway regulates Bcl-xL, and the external stimulation with CD40L does not induce higher expression of this anti-apoptotic protein in REC-1 cells, most likely due to the already high basal activity of this pathway.

### CD40L-mediated alternative NFκB signaling in MCL cell lines is independent of the BCR pathway

**Fig. 3:**
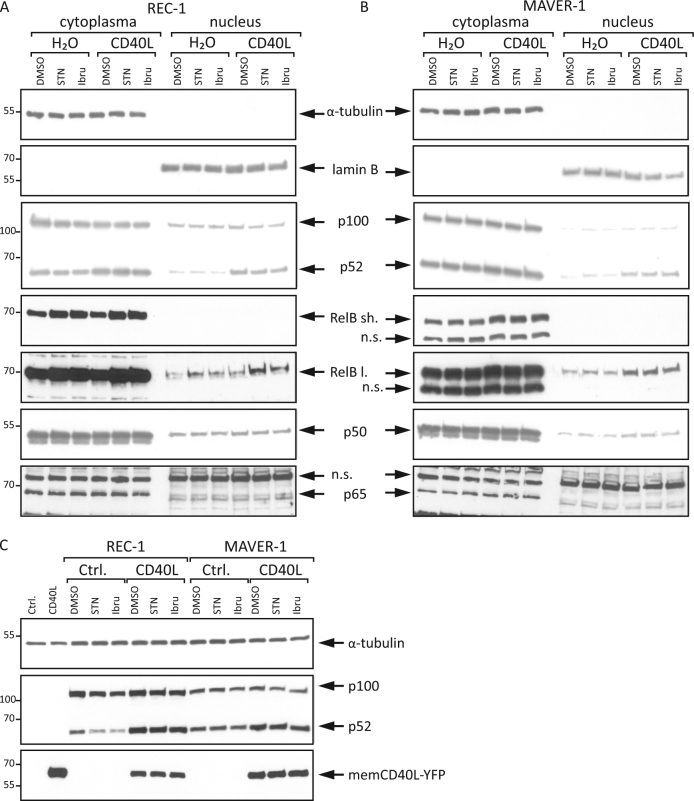
CD40L activates alternative NFκB signaling independently of the BCR pathway The MCL cell lines REC-1 **a** and MAVER-1 **b** were preincubated with CD40L (100 ng/ml) or H_2_O for 18 h and subsequently treated with either sotrastaurin (STN, 3 μM), ibrutinib (Ibru, 400 nM) or DMSO as a control for an additional 24 h. Cytoplasmic and nuclear protein extracts were analyzed by Western blot for the indicated proteins; “sh.” stands for a short and “l.” for a long exposure time for RelB detection. **c** L-929 cells were transfected with a CD40L expression plasmid or a control plasmid and co-cultured with REC-1 and MAVER-1 cells for 18 h. Subsequently, the cells were treated with sotrastaurin (3 µM), ibrutinib (400 nM) or DMSO for an additional 24 h, and whole cell lysates were analyzed by Western blot

To decipher the potential effect of this signaling pathway on drug resistance, we prestimulated the BCR inhibitor-sensitive cell line REC-1 and the inhibitor-resistant cell line MAVER-1 with CD40L overnight to activate the alternative NFκB pathway. Subsequently, the cells were treated with two inhibitors: ibrutinib or sotrastaurin. Western blot analysis of cytoplasmic and nuclear protein fractions revealed that CD40L-induced p100 processing to p52 and its nuclear translocation were influenced neither by the PKC inhibitor sotrastaurin nor by the BTK inhibitor ibrutinib (Fig. 3a, b). The same effect was detectable for the enhanced expression and nuclear levels of RelB after CD40L treatment in REC-1 cells. In MAVER-1 cells, this effect on RelB was also obvious, whereas the effect on p100/p52 was preeminent in REC-1 cells. This difference might be due to the higher basal levels of the alternative NFκB pathway activity in MAVER-1 cells. Furthermore, CD40L treatment had no major effect on the NFκB transcription factors p50 and p65 (Fig. 3a, b), which are regulated by the classical NFκB pathway^27^. This result indicates a more prominent role of CD40L on the alternative than on the classical NFκB pathway in this experimental setting. Additionally, L-929 cells were transiently transfected with a CD40L expression plasmid and co-cultivated with REC-1 and MAVER-1 cells (Fig. 3c). This microenvironment-mimicking experiment showed similar results as stimulation with recombinant CD40L. The induction of p100 processing to p52 was not influenced by inhibition of the BCR pathway. Therefore, both cell lines responded to CD40 stimulation with accelerated activity of the alternative NFκB pathway, and this signaling was neither influenced by the BTK inhibitor ibrutinib nor by the PKC inhibitor sotrastaurin.

### Activation of the alternative NFκB pathway by recombinant CD40L induces resistance to BCR inhibitors in REC-1 cells

**Fig. 4:**
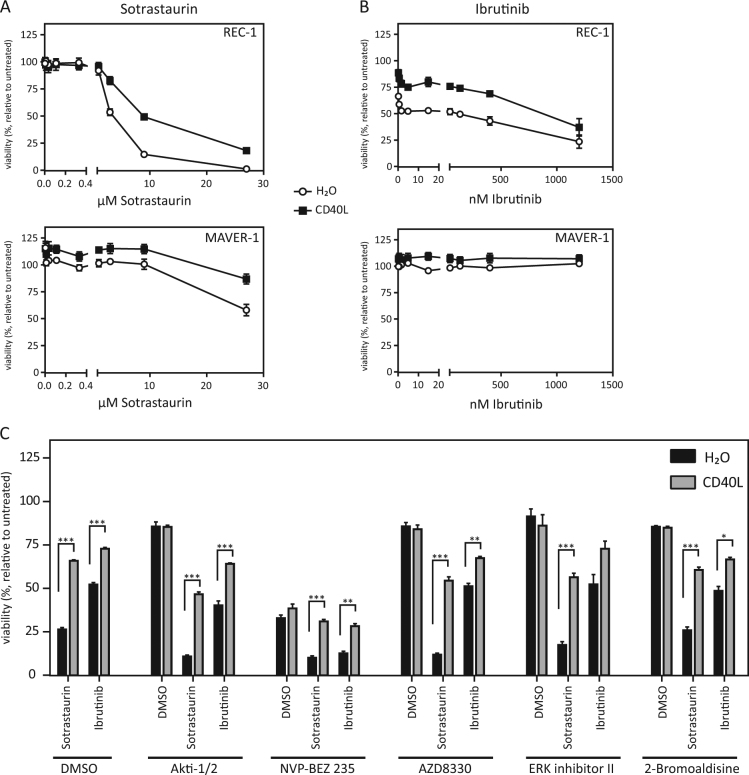
CD40L induces drug resistance in MCL cell lines. **a**, **b** Viability assays were performed after prestimulation of REC-1 and MAVER-1 cells with CD40L (100 ng/ml, 18 h) or H_2_O as a control and additional treatment with sotrastaurin (**a**, 3 µM) or ibrutinib (**b**, 400 nM) for 48 h. Viability was determined with the MTT assay. **c** REC-1 cells were treated with various drugs (Akti-1/2, 1 µM; NVP-BEZ 235, 25 nM; AZD8330, 500 nM; ERK inhibitor II, 10 µM; 2-Bromoaldisine, 1 µM) or DMSO for 20 min before stimulation with CD40L (100 ng/ml, 18 h) or H_2_O as a control and subsequent treatment with sotrastaurin (3 µM), ibrutinib (400 nM) or DMSO for additional 48 h. Viability was determined with the MTT assay (*p < 0.001; **p < 0.0001; ***p < 0.00001)

Activation of the alternative NFκB pathway in MCL cell lines results in nuclear translocation of the transcription factors p52 and RelB (Fig. 3a, b). Although little is known specifically about CD40L-induced p52/RelB target genes, it is established that these transcription factors play a major role in B cell development, survival and homeostasis (reviewed in^29^). Therefore, we questioned whether CD40L-induced signaling in MCL cells causes drug resistance. Viability assays revealed a protective effect of CD40L prestimulation on BCR inhibitor treatment (Fig. 4). Profoundly, this effect was detectable and significant in the sensitive cell line REC-1 for both inhibitors at clinically relevant concentrations (sotrastaurin 3 µM: *p* < 0.0001 and ibrutinib 400 nM: *p* < 0.0001) but also in the resistant cell line MAVER-1 at nonphysiologically high sotrastaurin concentrations (27 µM: *p* < 0.001) (Fig. 4a). These data argue for a protective role of CD40L-mediated alternative NFκB signaling leading to drug resistance in MCL.

CD40 is known to activate also various other pathways than the NFκB pathways^30^. Therefore, we studied the effects of inhibitors of diverse pathways on the CD40L mediated resistance to the BCR inhibitors in REC-1 cells (Fig. 4c). Apart from NVP-BEZ 235, a dual PI3K and mTOR inhibitor, the other inhibitors (Akti-1/2, a selective inhibitor of Akt1 /Akt2; AZD8330 a selective MEK1/2 inhibitor; ERK inhibitor II, an inhibitor of ERK1 and ERK2; 2-Bromoaldisine, an inhibitor of the Raf/MEK-1/MAPK cascade) alone show little toxicity. The combination of the inhibitors with BCR inhibitors showed only slight additive toxic effects. Interestingly, CD40L prestimulation rescued REC-1 cells from the sotrastaurin and ibrutinib mediated toxicity even in combination with the different inhibitors. Regarding Akti-1/2, AZD8330, ERK inhibitor II and 2-Bromoaldisine the rescue effect of CD40L is hardly influenced by the additional pathway inhibition and the rescue effect is still highly significant, with only one exception (ERK inhibitor II in combination with ibrutinib). Also, the REC-1 cells treated with NVP-BEZ 235 are in part rescued from the toxicity induced by the BCR inhibitors. This supports the protective function of the alternative NFκB pathway against BCR inhibitors in MCL cells.

## Discussion

Targeting the BCR pathway is a new and promising approach to treat MCL, and ibrutinib as a clinically used drug targeting this pathway in MCL shows favorable response rates^31,32^. Unfortunately, subsequent studies have revealed that some patients develop resistance upon ibrutinib treatment and relapse^7,33^. Therefore, efforts have been made to develop second-generation BCR inhibitors and to decipher the formation of drug resistance in MCL. In our own and other studies, it was shown that the MAVER-1 cell line is resistant to the two inhibitors ibrutinib and sotrastaurin, whereas the cell line REC-1 is sensitive^9,25^. Rahal et al. further postulated an important role of the alternative NFκB pathway in BCR inhibitor resistance for MCL cells, as, for example, the resistant MAVER-1 cell line harbors a biallelic deletion of TRAF3^9^. TRAF3 has an inhibitory function in regulating the alternative NFκB pathway^34^. Apart from specific mutations, microenvironmental factors can also influence the activity of the alternative NFκB pathway. Particularly, for MCL, stromal cells have been shown to activate this signaling pathway^35^. Therefore, we questioned the role of external signaling on the alternative NFκB pathway in MCL and its effect on drug responses.

Our data illustrate the high basal activity of the alternative NFκB pathway in MAVER-1 cells compared to that in REC-1 cells, most likely caused by the TRAF3 deletion in MAVER.1 cells. In addition, MAVER-1 cells show lower p100 levels than REC-1 cells, arguing not only for a higher activity of the alternative NFκB pathway but also for a lower activity of the basal classical NFκB signaling pathway, as p100 is a target of classical NFκB signaling^22,27^. Additionally, Supplementary Figure 1 illustrates the reduction of p100 levels in the context of CD40L treatment and additional inhibition of the classical pathway, underlining the activating role of CD40L on alternative NFκB signaling in MCL cells. Furthermore, MAVER-1 cells are less dependent on classical NFκB signaling, leading to the assumption that alternative NFκB signaling in part may substitute for classical signaling in MCL cells.

To analyze the microenvironmental effect on the activity of the alternative NFκB pathway in MCL, we chose to examine CD40L. All MCL cells, as other B cells, express its receptor CD40^36^, and CD40L is expressed not only on T cells but also on other cell types^37,38^, which are common in the MCL microenvironment. Therefore, the CD40L-CD40 interaction and the CD40L-rich microenvironment play a part in the pathogenesis of MCL^39^. In addition, Chiron et al. previously described the role of CD40 stimulation on the activity of NFκB signaling in MCL^20,28^. In our experiments to stimulate CD40, we chose a highly active recombinant oligomer from Enzo, which mimics the in vivo-occurring membrane-assisted aggregation of CD40L. Our study showed a significant proliferative effect of CD40L stimulation on REC-1 cells and (to a lesser extent) on MAVER-1 cells. These data are contradictive to reports describing an antiproliferative effect of CD40L on MCL cell lines^40^ but are in line with other studies describing the CD40-system^15,39^. Furthermore, CD40L stimulation induced alternative NFκB pathway signaling in both cell lines. This activation rescued REC-1 cells from IKK2 inhibition-mediated toxicity and is therefore independent of signaling via CD40 on the classical NFκB pathway.

Because Rahal et al. previously postulated the possibility of activating mutations of alternative NFκB pathway members to substitute for dependency on BCR signaling, these data indicate the potential of microenvironmentally induced alternative NFκB signaling to substitute for classical NFκB signaling in dependent cells.

The CD40L-mediated activity of the alternative NFκB pathway was obvious in both cell lines, as shown by enhanced nuclear p52 and RelB levels. Although MAVER-1 cells possess higher basal levels of alternative NFκB signaling activity due to *TRAF3* deletion, external ligand stimulation could push this signaling to a higher level. However, in both cell lines the BCR inhibitory drugs sotrastaurin and ibrutinib had no major effect on the transcription factor p52, neither at the basal nor at the induced level. Only RelB levels were slightly enhanced after inhibitor treatment in REC-1 cells which might be caused by an unknown inhibition mediated feedback loop. Similar but even more robust effects regarding p100 processing to p52 were detectable in the microenvironment-mimicking co-culture experiment. In sum, these data indicate that alternative NFκB signaling pathway activity is independent of BCR signaling in MCL cells. With regard to viability, it was clear that CD40L stimulation protected REC-1 cells from BCR inhibitor-mediated toxicity, especially at clinically relevant concentrations, whereas no major effect was seen in the resistant cell line MAVER-1. This effect seems not to be dependent on the anti-apoptotic protein Bcl-xL or induced classical NFκB signaling. Additionally, inhibition of various other pathways, which can be influenced by CD40 stimulation, had no impact on the CD40L mediated rescue of REC-1 cells and therefore supports the protective role of alternative NFκB pathway activity against BCR inhibitors in MCL.

We also analyzed the ability of TNF ligand superfamily member 13b (BAFF) to activate the alternative NFκB pathway in the studied MCL cell lines. Unfortunately, stimulation with BAFF did not robustly induce p100 processing to p52 nor did it clearly rescue REC-1 cells from TPCA-1 or BCR inhibitors-mediated toxicity (data not shown). Experiments to generate REC-1 cells with a silenced alternative NFκB pathway in order to strengthen the functional role of this pathway in drug resistance were unsuccessful and further studies are needed to decipher the substantial role of the alternative NFκB pathway in MCL.

In conclusion, the data presented in this study argue for the protective potential of microenvironmentally mediated activation of the alternative NFκB pathway in MCL cells against BCR signaling-associated drugs, which might represent a physiologic niche for MCL relapse. Additionally, these data provide evidence for the potential of the alternative NFκB pathway as a possible therapeutic target in MCL.

## Materials and methods

### Cell lines reagents and antibodies

REC-1, MAVER-1, and L-929 cell lines were purchased from Deutsche Sammlung von Mikroorganismen und Zellkulturen (DSMZ, Braunschweig, Germany) and were cultured in RPMI1640 (Thermo Fisher Scientific, Waltham, MA, USA) with 10% (REC-1, L-929) or 20% (MAVER-1) heat-inactivated fetal bovine serum (PAN Biotech, Aidenbach, Germany and Thermo Fisher Scientific) supplemented with 2 mmol/l l-glutamine (PAN Biotech). All cell lines were cultured at 37 °C with 5% CO_2_ and were regularly tested for the absence of mycoplasma infection with the Venor GeM OneStep kit (Minerva Biolabs, Berlin, Germany).

MEGACD40L (referred to as CD40L) and 2-Bromoaldisine were purchased from ENZO Life Sciences (Lörrach, Germany). MG132 was purchased from Sigma-Aldrich (Munich, Germany), and TPCA-1, ibrutinib and sotrastaurin were purchased from Selleck Chemicals (Absource Diagnostics, Munich, Germany). NVP-BEZ 235 was purchased from Hycultec (Beutelsbach, Germany) and AZD8330 was purchased from Biozol (Eching, Germany). Primary antibodies for PARP (#9532), NIK (#4994), β-actin (#4970), CD40L (#15094), Bcl-xL (#54H6) and secondary horseradish peroxidase (HRP)-conjugated anti-rabbit IgG-HRP antibody (#7074) were purchased from Cell Signaling Technologies (Danvers, MA, USA). The primary antibodies for lamin B (#sc-6216), p65 (#sc-71677), p50 (#sc-7178) and RelB (#sc-226) were from Santa Cruz Biotechnology (Dallas, TX, USA). The p100/p52 (#05–361) antibody, ERK inhibitor II and Akti-1/2 were purchased from Merck Millipore (Darmstadt, Germany), and α-tubulin (DM1A) was purchased from Thermo Fisher Scientific. Secondary HRP-conjugated anti-mouse (#P0260), anti-goat (#P0160), and anti-rabbit (#P0448) antibodies were purchased from Dako (Glostrup, Denmark).

### Viability assay

Cells were seeded into 96-well plates and stimulated as indicated in the various figure legends. After the specific treatments, metabolic activity was measured with the cell proliferation kit I (3-(4,5-dimethylthiazol-2-yl)-2,5-diphenyltetrazolium bromide; MTT) (Roche Diagnostics, Mannheim, Germany) in accordance with the manufacturer’s instructions. Cell viability was calculated as the percentage of cells either treated with dimethyl sulfoxide (DMSO, Carl Roth, Karlsruhe, Germany) or untreated (100%) and background staining in dead cells (0%) was measured following treatment with H_2_O_2_ (Merck Millipore Darmstadt, Germany). DMSO treatment was performed in the equivalent dilution as the highest corresponding reagent dilution in the individual experiment, and the error bars represent +/− standard error of the mean (SEM). Combined data are shown from two or three identical individual experiments.

### Co-culture with CD40L-expressing cells

L-929 cells were transiently transfected with Lipofectamin3000 according to the manufacturer´s instructions with a CD40L expression plasmid (human full-length CD40L-pEYFP-C1) or a control plasmid (pEYFP-C1) over-night. Prof. Dr. Harald Wajant kindly provided both plasmids. The next day, the cells were seeded and co-cultured with REC-1 and MAVER-1 cells. After 18 h of co-culture, the cells were treated with sotrastaurin, ibrutinib or DMSO as a control. After an additional 24 h, the cells were harvested, and whole cell lysates were prepared. The transfection efficiency was verified by analyzing the membrane CD40L expression by Western blot.

### Western blot

Cells were stimulated as indicated in the individual figure legends and subsequently washed with PBS. Nuclear and cytoplasmic protein fractions were isolated by use of the CelLytic NuCLEAR Extraction Kit from Sigma-Aldrich (Munich, Germany) according to the manufacturer’s instructions. Whole cell lysates were prepared by lysing cell pellets for 20 min on ice in lysis buffer (20 mM Hepes; 350 mM NaCl; 1 mM MgCl_2_; 0.5 mM EDTA, pH 8.0; and 0.1 mM EGTA, pH 8.0). After centrifugation, the protein concentrations were measured from the supernatant with the Bradford assay, and the lysates were supplemented with loading buffer (100 mM Tris; pH 6.8; 20% glycerol; 8% SDS; 10% beta-mercaptoethanol; and 0.01% bromophenol blue) and boiled to 96 °C for 5 min. Samples were then loaded for sodium dodecyl sulfate polyacrylamide gel electrophoresis, and the proteins were transferred to nitrocellulose membranes and checked by Ponceau S (Sigma-Aldrich) staining. Blots were subsequently incubated for 1 h with Tris-buffered saline containing 0.1% Tween 20 and 5% milk powder and incubated with primary and HRP-conjugated secondary antibodies. The antigen-antibody complexes were visualized with SuperSignal West Pico Chemiluminescent Substrate (Thermo Fisher Scientific, Waltham, MA, USA) and Amersham Hyperfilm ECL (GE Healthcare, Freiburg, Germany). Different proteins were in part visualized on separate but identical blots.

### Statistical analysis

Statistical analysis was performed using GraphPad Prism 6 (GraphPad Software, Inc., La Jolla, CA, USA) to analyze the significance of viability changes after CD40L prestimulation (Fig. 2b) with multiple *t*-tests (* *p* < 0.001; **p < 0.0001; ***p < 0.00001).

## Electronic supplementary material

Supplementary Figure Legend

Supplementary Figure 1
