# Supplementary material for: CD40L mediated alternative NFκB-signaling induces resistance to BCR-inhibitors in patients with mantle cell lymphoma
Source: Cell Death Dis. 2018 Jan 24;9(2):86. doi: 10.1038/s41419-017-0157-6 (PMC5833745; doi:10.1038/s41419-017-0157-6)
Supplement: Supplementary file 1 — Supplementary Figure Legend [file 41419_2017_157_MOESM1_ESM.docx]

**CD40L mediated alternative NFκB-signaling induces resistance to BCR-inhibitors in patients with mantle cell lymphoma**

**Supplementary Figure 1 Legend**

Bcl-xL expression in MCL cell lines. REC-1 and MAVER-1 cells were treated with TPCA-1 (2.5 µM) or DMSO for 30 min, and then CD40L (100 ng/ml, 18 h) or H_2_O was added. Whole cell lysates were analyzed by Western blot.
