# Supplementary figures and images for: CD40L mediated alternative NFκB-signaling induces resistance to BCR-inhibitors in patients with mantle cell lymphoma
Source: Cell Death Dis. 2018 Jan 24;9(2):86. doi: 10.1038/s41419-017-0157-6 (PMC5833745; doi:10.1038/s41419-017-0157-6)

Supplementary Figure 1

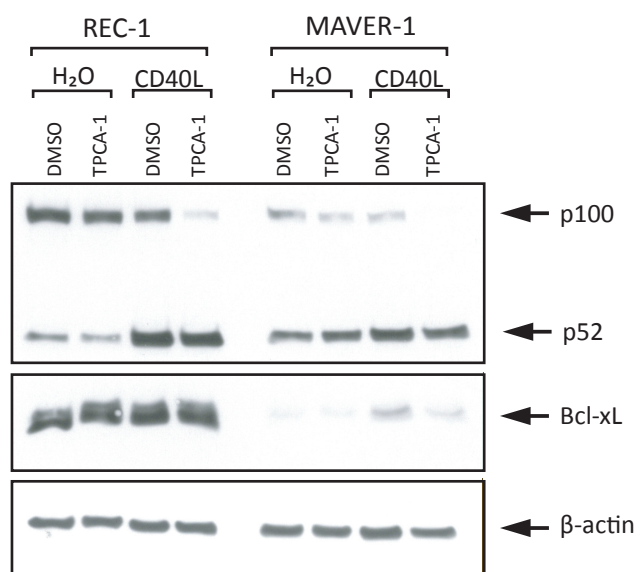

Supplement: Supplementary file 2 — Supplementary Figure 1 [file 41419_2017_157_MOESM2_ESM.pdf]
